# Access to European Union Agencies: Usual Suspects or Balanced Interest Representation in Open and Closed Consultations?

**DOI:** 10.1111/jcms.12991

**Published:** 2019-12-29

**Authors:** Sarah Arras, Jan Beyers

**Affiliations:** ^1^ Department of Political Science University of Antwerp Antwerp

**Keywords:** EU agencies, consultations, interest groups, regulatory politics

## Abstract

To facilitate stakeholder representation, European Union (EU) agencies use a range of procedures, including closed consultation or advisory committees and open or public consultations. For analysing what kind of stakeholders gain access to advisory committees, we compare these two particular procedures. Two theoretical perspectives guide this analysis. The first is a resource‐based account, which emphasizes informational needs and leads to the expectation that not only regulated interests but also EU‐level associations and European Commission expert group members will gain representation through closed consultations. The second is a norm‐based perspective that stresses the importance for agencies to establish a credible reputation, leading them to balance interest representation. A systematic comparison of stakeholders represented in agency committee with those participating in open consultations demonstrates that regulated interests have no systematic advantage in gaining access to closed consultations. Instead, closed consultations may diversify interest representation and facilitate the involvement of non‐business interests.

## Introduction

In November 2015, advice given by the European Food Safety Authority (EFSA) to the European Commission (EC) ignited public outcry. The EFSA had concluded that glyphosate – the active substance in Monsanto's top selling pesticide, Roundup – is unlikely to cause cancer in humans and therefore proposed a higher ceiling for the amount of residue considered safe for human consumption (EFSA, 2015). However, environmental non‐governmental organizations (NGOs) had been calling for a ban on glyphosate since the International Agency for Research on Cancer (IARC) had said that glyphosate is carcinogenic to humans. The initial discussion on glyphosate's carcinogenic properties turned into a debate about the EFSA's close contacts with the industry, the potential risks to the independence of its scientific advice, and the agency's credibility as an independent regulator more generally.

This example illustrates how relations with private interests can pose a potential threat to European Union (EU) agencies' autonomy and credibility (Arras and Braun, 2018; Borrás et al., 2007; Carpenter and Moss, 2014; Thiel, 2014). As these agencies are non‐majoritarian bodies with far‐reaching regulatory competences, some engagement with societal interests can be useful to fulfil informational needs, ensure compliance, and uphold a credible reputation (Borrás et al., 2007; Braun, 2012; Busuioc and Lodge, 2016; Carpenter, 2001; Coglianese et al., 2004; Furlong and Kerwin, 2005; Koop, 2014; Thiel, 2014). However, interactions between independent agencies and stakeholders, especially regulated business interests, imply a risk of autonomy loss and may lead to regulatory outcomes favouring special interests (Carpenter and Moss, 2014; Stigler, 1971). Agencies may reduce this risk by institutionalizing a more balanced interest representation and facilitating the consultation of non‐regulated and non‐business interests such as consumer organizations, environmental NGOs, or patient groups (Kwak, 2014). This paper contrasts two consultation procedures, namely advisory committees (a *closed‐access procedure*) and public consultations (an *open‐access procedure*). An important question is whether open and closed procedures foster the involvement of a diverse set of stakeholders and to what extent those who gain access to advisory committees (committees hereafter) are representative for a wider range of interests.

Establishing committees and strategically granting access to actors with an interest in agency policymaking are powerful tools for agencies to manage their interactions with the broader environment. The selection of committee members reflects an agency's view of who is relevant to involve in regulatory policymaking. Actors who enjoy access have an insider position and are better informed. As a result, they have more opportunities to influence regulatory outcomes. Committee members are involved in the regulatory process at an early stage and often provide feedback on draft documents before these become publicly available (Binderkrantz et al., 2015; Fraussen et al., 2014; Rasmussen and Gross, 2015). Moreover, face‐to‐face meetings with agency officials as well as with other committee members represent important networking opportunities (Binderkrantz et al., 2015/Binderkrantz et al., 2016). Given the role committee members may play in developing regulatory outcomes, it is important to know who is included and whose interests are represented in these closed consultations.

The paper starts with a short literature review on why and how EU agencies interact with societal interests. This leads us to identify two key mechanisms for involving societal interests, one that is resource‐based and another that is norm‐based. Based on this we develop three hypotheses explaining how agencies select committee members. These hypotheses are tested using a novel database that combines data on committee members with evidence on open consultation. The results show that business interests, especially representatives from regulated business interests – the industries which are directly targeted by agency regulations – are strongly represented in committees. However, despite the prominent position of regulated industries, we cannot conclude that agencies systematically favour them to the disadvantage of non‐business interests. For instance, non‐business interests, NGOs, and labour unions, especially in comparison with their participation in open consultations, gain quite substantial access in closed consultations. Another relevant finding is that EC expert group membership strongly predicts committee access, which implies that committees tend to privilege groups that already have an insider position during earlier stages of the policymaking process.

## Stakeholder Consultation Procedures

Regular and repeated interactions between regulatory agencies and societal interests are somewhat puzzling given the overall rationale behind the establishment of agencies, especially when regulated industries are concerned. The reason for delegating to agencies is that regulatory outcomes are assumed to be more credible when they are handled by unelected experts and insulated from politics, and when private interests are kept at a distance (Barkow, 2010; Majone, 1999; Wonka and Rittberger, 2010). Complete independence, however, is hard to realize in practice and, if it did exist, could lead to suboptimal outcomes and ineffective regulation. Indeed, for agencies it is difficult to consider market specificities and anticipate consequences for an industry if they do not have access to information available within the industry itself. For instance, during one of the interviews we conducted within the framework of this project, an EU agency official described the reason for involving societal interests as follows:

‘We do not want to be the authority that lives in the ivory tower that invents regulation without knowing exactly what kind of impact it will have. It helps us to avoid the mistake when you make a regulation and then, afterwards, you have to change it because nobody is able to comply with this regulation’ (EU agency official, 11/12/2015). 1The quotations in this article come from interviews with stakeholder relations officers in EU regulatory agencies that were conducted by the authors in 2015–2016 for a research project on how EU agencies consult with organized interests (Arras and Braun, 2018).


In analysing how agencies interact with stakeholders, we start from two goals that agencies need to reconcile, namely establishing effective and legitimate policies. First, in order to draft and implement efficient and effective regulatory policies, agencies need resources – mostly information and expertise, but also support – which in many instances can be supplied by organized interests. This first goal fits within a classic resource exchange perspective, often used by interest group scholars to explain why some organized interests enjoy access to policymakers. In this view, policymakers need resources – policy relevant expertise and information – which are supplied by organized interests in the hope of obtaining access and policy influence (Binderkrantz et al., 2015; Bouwen, 2002; Braun, 2012; Rasmussen and Gross, 2015). Second, agencies seek to preserve their autonomy because having the reputation of being an independent and impartial regulator is a constituent feature of their legitimacy. This goal reflects the notion that the long‐term survival of agencies depends on whether the environment perceives them as trustworthy and reliable, which makes agencies legitimacy‐seeking organizations (Hannan and Freeman, 1989). Legitimacy concerns imply that, while interacting with societal interests, agencies, as well as their principals, avoid situations that make them too dependent on one single type of interest (for instance, regulated business). Instead, fostering a balanced representation of a diverse array of societal interests may strengthen an agency's reputation as an autonomous and legitimate authority.

Scholars often consider facilitating the involvement of a broad array of societal interests, and not only specialized groups which are successful at providing expert information, as appropriate and legitimate administrative behaviour (Furlong and Kerwin, 2005; Gornitzka and Sverdrup, 2015; Koop, 2014; see also March and Olsen, 1989). In short, EU agencies actively involve stakeholders due to a normative belief that some form of participatory democracy contributes to the agencies' overall legitimacy (Kohler‐Koch and Quittkat, 2013). In doing this, agencies are involved in a delicate balancing act. On the one hand, complete isolation from regulated business interests may, due to the absence of critical information, lead to ineffective regulation. On the other hand, an involvement that is too close can threaten agencies' autonomy and cause regulatory capture (Carpenter, 2001/2010; Carpenter and Moss, 2014; Stigler, 1971). In order to cope with this tensions, various scholars have observed that regulatory agencies may try to consult with a diverse set of interests and not only with those businesses that need to be regulated; hearing different voices may soften the dependence on one type of interest (Arras and Braun, 2018; Braun, 2012; Yackee and Yackee, 2006).

Importantly, consultation procedures may vary considerably in terms of inclusiveness (*closed* versus *open*), and more precisely with respect to the types of interests that gain representation (for instance, business and/or citizen interests) (Beyers and Arras, 2019; Pedersen et al., 2015; Quittkat and Kotzian, 2011; Van Ballaert, 2017). *Open* procedures refer to public consultations and rely largely on the bottom‐up mobilization of societal interests (Halpin, 2011; Pagliari and Young, 2016; Rasmussen, 2015; Yackee, 2014; Young and Pagliari, 2015; Quittkat, 2011). When an agency wants to establish a new policy or change some existing policy, it launches an open call, inviting all stakeholders (individual citizens, companies, or organizations), to submit their opinions and supply relevant information. Usually such consultations and the submitted opinions are processed via a website portal and may involve a list of specific closed and/or open questions. In the final stage of the consultation, the agency publishes a report in which it summarizes the submitted opinions and clarifies how and to what extent the proposed policy was modified in view of the received information (Quittkat, 2011; Yackee and Yackee, 2006). Although sometimes a particular audience is addressed in the consultation call, a key feature is that in principle anyone can participate. However, the decision to do so lies entirely with the stakeholders themselves.

Advisory committees are *closed* procedures. They are permanent bodies within the agency, in which a limited number of stakeholders – interest groups, but also independent experts or individual companies – hold a seat for a longer period of time (Binderkrantz et al., 2015; Fraussen *et al*., 2014b; Gornitzka and Sverdrup, 2015; Rasmussen and Gross, 2015). The key difference with open consultations is that policymakers control who can participate in the consultation. Sometimes, a specific regulation stipulates which actor types should be represented, but, in general, the agencies themselves have some discretion about who will become a member of a committee. Given the type of format, the number of stakeholders involved via committees is usually lower than in public consultations, but the setting allows for repeated interactions and deliberation among stakeholders (Braun, 2012; Carpenter, 2001; Carpenter, 2010). As agency leaders enjoy some discretion regarding whom they consult, they can use this mechanism to diversify interest representation and, in doing so, prevent excessive dependence on one type of stakeholder.

While this study is one of the first to focus on stakeholder representation in the context of EU regulatory agencies (but see Beyers and Arras, 2019; Arras and Braun, 2018; Borrás et al., 2007; Koop, 2014; Pérez Durán, 2018/Pérez Durán, 2019), some research has examined who gains access to committees and how such insiders are representative for a wider population of mobilized interests (Binderkrantz et al., 2015; Fraussen and Beyers, 2015; Mahoney, 2004; Rasmussen and Gross, 2015). In order to draw robust conclusions regarding the representation of societal interests, contrasting evidence on how bottom‐up mobilization patterns of open consultations correspond with the insider status that stakeholders enjoy closed consultations is highly relevant. One important question is to what extent the agency leadership uses its discretion to facilitate the diversification of interest representation by selecting non‐regulated interests. Or more generally, to what extent does it make a difference for consultation practices whether or not an agency uses closed or open procedures? On the one hand, we can expect the selection of committee members by agency officials to reflect the distribution of the stakeholders mobilized during open consultations. Given the uncertainty that characterizes much regulatory policymaking, agency officials cannot predict in advance and in a precise manner what type of information or expertise will be most valuable and who will be able to supply this. Therefore, comparing the profile of committee members with a broader array of mobilized interests is theoretically relevant. Participation in open consultations signals a policy interest and demonstrates the stakeholders' preparedness and ability to share information with policymakers. Evidence on the overall set of mobilized interests – for instance, during open consultations – is the first important piece of information agency officials may rely on when selecting committee members.

Hence, our null‐hypothesis is that the distribution of stakeholders participating in open consultations does not differ significantly from the set of actors involved in closed consultations. Rejecting the null‐hypothesis implies that some theoretically relevant variables predict significant differences between the two consultation forms, meaning that the adopted consultation form establishes a substantial difference in terms of who is consulted. More generally, we hypothesise that the insider position of a stakeholder in a committee will largely be shaped by its status in the eyes of policymakers, which relates to the overall potential to provide valuable resources, in particular policy expertise and societal legitimacy. As Grossmann argues, ‘policymakers rely on basic signals about organizations that allow them to make comments like “they have credibility” and “they're known players”, without fully processing how they arrived at these judgments’ (Grossmann, 2012, p. 165; see also Fraussen and Beyers, 2015). In addition to participating in public consultations, agencies use other shortcuts to establish who is valuable in terms of policy know‐how or political support. Among these shortcuts are interest group type, the access to EC expert bodies, and the extent to which a group represents an encompassing interest, and our research specifically focuses on these.

## Gaining Access to Closed Consultations

The need for expert information results from agencies' responsibility to implement regulatory policies which are often of a highly technical nature (such as chemicals or financial markets) (Coglianese et al., 2004; Pagliari and Young, 2016). Somewhat in contrast to legislative politics, political information (for instance, the amount of support a policy enjoys), might be less pivotal for unelected agency bureaucrats who are insulated from electoral politics (De Bruycker, 2016). Hence, information exchanges between agencies and societal interests are primarily expertise‐based. Expertise can be (partially) generated by in‐house experts, as the logic of ‘delegation to experts’ implies (Majone, 1999), but agency officials often rely on external actors to fulfil their informational needs. This is especially the case for EU‐level agencies whose in‐house resources are limited given the scope of their competences and limited staff resources. As one EU agency official clarified during one of our interviews:

‘It is clear that, especially when you have 140 staff members, you cannot know everything, all the nuances that are happening across the EEA (European Economic Area). Because how would you know about what is happening in Bulgaria if you are sitting in [agency location]?’ (EU agency official, 07/05/2015).

Technical knowledge on how regulatory policies affect specific markets (for instance, producers, retailers, traders) is strongly controlled by regulated business interests, namely, the industries that are directly targeted by agency regulations. Their natural informational advantage stems from an acquaintanceship with companies that, as a by‐product of their everyday economic activities, possess relevant policy expertise (Barkow, 2010; Bouwen, 2002; Pagliari and Young, 2014). Moreover, the fact that they are directly affected by the potential costs of regulation makes them highly motivated to develop expertise, engage in collective action, and lobby regulatory agencies (Olson, 1965; Pagliari and Young, 2014). For instance, more stringent regulations in the financial sector directly affect financial service providers, which triggers the mobilization of regulated business interests in this field. Regulatory policies, however, also affect non‐business interests, such as consumers, or other more indirectly affected business interests. Less stringent (or non‐existent) regulations may cause negative externalities for consumers, and some regulations may increase production costs in other industries. For example, adopting stricter regulations with respect to lending could constrain the capability of small and medium enterprises to attract risk capital. The diffuse nature of many positive/negative externalities means that interests which are not directly regulated – for instance, consumers or other businesses – face higher barriers to developing knowledge, mobilizing, and influencing regulatory outcomes.

This resource‐based approach presumes that agencies will select representatives who are most capable of supplying technical expertise that helps agencies to produce apolitical and Pareto efficient outcomes, or who are at least perceived to be such (Majone, 1999). However, the abovementioned glyphosate debate illustrates that EU agencies deal with technically complex issues where knowledge is contested, and regulations create winners and losers. As stated, agency officials are also driven by a norm‐guided view – a logic of appropriateness – which instructs them to stay in touch with a broader range of stakeholders (Gornitzka and Sverdrup, 2015; March and Olsen, 1989). Showing that a diverse set of interests has been consulted can foster an agency's reputation as an autonomous actor, contribute to its legitimacy, and improve its brokerage potential (Braun, 2012; Carpenter, 2001/2010; Moffitt, 2010). In contrast, interacting with a limited set of stakeholders, or more specifically, with only regulated business interests, can threaten agencies' autonomy and increase the likelihood of capture (Carpenter and Moss, 2014; Kwak, 2014). Facilitating a balanced and diverse representation of interests is not entirely at odds with an informational resource‐based account. Compared to regulated business interests, other interests – for instance, other business groups or consumer groups – are more inclined and motivated to develop expertise on positive and negative externalities, which makes them also a relevant alternative informational source. These reflections lead to two competing hypotheses:

Hypothesis 1a Stakeholders representing regulated business interests are more likely to be selected for committees compared to actors representing other interests.

Hypothesis 1b Stakeholders representing regulated business interests are not more likely to be selected for committees compared to actors representing other interests.

In addition to information on organization type, in other words, whether the stakeholder is a representative of the regulated business interests or not, agency officials use other shortcuts, pointing at the extent to which an organized interest will be able to supply relevant knowledge. One such shortcut is whether or not a stakeholder enjoys an insider status in other EU arenas. Given the role EC expert groups play in the legislative phase, members of these expert groups may have expertise that can be useful for EU agencies that design regulatory policies which implement EU legislation (Gornitzka and Sverdrup, 2011/Gornitzka and Sverdrup, 2015; Rasmussen and Gross, 2015). In this way, the status of belonging to an EC expert group signals a reputation of expertise and experience. Therefore, organized interests with a seat in one of the EC expert groups will gain easier access to closed consultations. Access is thus cumulative, with access in one arena spilling over to access in other arenas, a phenomenon similar to the so‐called Matthew effect (Binderkrantz et al., 2015; Merton, 1968).

Hypothesis 2 Interest groups with a seat in an EC expert group are more likely to be selected for committees.

EU agencies organize closed consultations through establishing committees not only to obtain expertise but also to profit from the organizational capacity of interest groups, as this may ease the implementation process (Arras and Braun, 2018; Braun, 2013; Coglianese et al., 2004; Verbruggen, 2013). EU agencies need support from, or want to avoid systemic opposition from, organized interests who have to comply with a particular regulation or who are indirectly affected by agency decisions. By institutionalizing interactions with various societal interests via committees, agencies mobilize the organizational capacity of affected stakeholders in order to facilitate and monitor the implementation of agency regulations. Involving interested parties and providing opportunities for their input can foster ownership and compliance, leading to a more effective implementation (Martinez et al., 2013; Ottow, 2015). Closely involving stakeholders and mobilizing their organizational capacity is the essence of what Selznick described as the process of co‐optation (Selznick, 1948, pp.34–35) and is a traditional logic of corporatist social‐economic policymaking (Braun, 2012/2013; Molina and Rhodes, 2002). Important here is the intermediary function organized interests fulfil, as they can form a bridge between policymakers and a target population (Poppelaars, 2007). Since it is impossible for agencies with limited resources to interact with all relevant actors individually, the intermediation capacity of a small number of stakeholders who represent an encompassing constituency is crucial. This applies especially to encompassing EU‐level associations whose membership consists of nationally based interest associations (Bouwen, 2002; Kohler‐Koch and Quittkat, 2013; Quittkat and Kotzian, 2012).

Hypothesis 3 Compared to international, national, or sub‐national groups, EU‐level organizations are more likely to be selected for committees.

## Research Design

Out of all the EU regulatory agencies operational on 1 January 2015 (*n* = 16), nine were included in our analysis. The selection was based on two criteria: the agency should have closed consultations, i.e. committees with restricted access, and should organize open public consultations. This enabled us to assess to what extent the selected committee members are a cross‐section of the stakeholders mobilized via public consultations. For each agency, the founding regulation was scrutinized to see whether there were provisions about which types of stakeholder should be granted access (Arras and Braun, 2018; Pérez Durán, 2018). To perform legislative oversight, legislators aim for a certain balance of interests by institutionalizing the participation of non‐regulated interests (Kelemen, 2002; McCubbins and Schwartz, 1984). Therefore, legislators – the EC, the Council, and/or the EP – who establish agencies, may develop norms stimulating the consultation of non‐business groups such as consumer or patient organizations. Six of the nine founding regulations contain provisions on which stakeholders should be involved, and most agencies are expected to seek a balanced interest representation (for instance, producers and consumers in the case of EFSA) (Table 1A Online Appendix). The only exception is the EMA, where only patient organizations and healthcare professionals are included in committees, and the pharmaceutical industry has no direct formal access. The three remaining agencies – the EASA, the ECHA, and the ACER – have no provisions regarding whom to involve.

In the second step, we mapped all committee members based on the agency website. To allow comparison with stakeholders that could have an interest in influencing the agency – and thus could be interested in the work of committees or aspire to insider status – we created a second list with all participants in public consultations organized from 2013 to 2014. From the public consultation participants, public authorities (*n* = 208) were excluded, since the project focus is on non‐governmental stakeholders and their involvement in regulatory policymaking. 2Several EU‐level agencies grew out of EU‐wide regulatory networks consisting of national level regulators. National governments are key decision‐makers in EU legislative processes, and national regulators take an active part in implementing EU regulations (Egeberg and Trondal, 2017; Bunea and Thomson, 2015; Quittkat, 2011). The areas of aviation safety, medicine regulation, and aviation attract a substantial number of national regulatory authorities (Arras and Braun, 2018). For 41 stakeholders we could not find evidence on their organization type. 3Not all actors could be identified or coded. A small set of consultees submitted anonymized reports in public consultations and some actors that could not be identified and/or coded, either because they did not have a website or we could not find relevant information in other sources. This led to a list of 2280 unique actors, of which 278 actors belong to a committee. Of the identified stakeholders, 88% (*n* = 2016) were active vis‐à‐vis only one agency, either via a public consultation or via a committee membership. The stakeholder community that surrounds EU agencies is heavily fragmented, which is not surprising given the highly specialized policy fields in which these agencies are active; 10% of the identified actors (*n* = 264) had participated in two or more public consultations with different agencies or/and were a member of two or more committees from different agencies. The European Chemical Industry Council (CEFIC), for instance, had a seat on committees from three agencies: the ACER, the ECHA, and the EFSA. To account for the instances where we encountered multiple membership, the dataset was organized in a long format with multiple rows for actors linked to more than one agency.

Each identified stakeholder was coded based on information available on stakeholders' website. 4The coding scheme was developed on the basis of an existing codebook developed for various other research projects (on transnational advocacy, the INTEREURO‐project; see www.intereuro.eu). If no or insufficient information could be obtained from the organizational website, it was retrieved from other sources such as Wikipedia, Linkedin, and the EU's Transparency Register. In several instances, we were able to obtain relevant information from position papers submitted during the public consultations. In addition to the authors, three coders took part in the coding process. For improving validity and reliability, all codes were cross‐checked by different coders, and in cases of doubt, we organized meetings between coders to establish a final code.

To test if members of an EC expert group gain better access (Hypothesis 2), we coded whether or not each group belongs to an EC expert group by matching our dataset to the EC expert group register that is available on the official EC website. 5
http://ec.europa.eu/transparency/regexpert/ (retrieved 11 November 2015). For testing Hypothesis 3 regarding the organizational scope of stakeholders, we coded whether organizations represent an international, EU‐wide, national, or sub‐national constituency.

The variable stakeholder type consists of four distinct categories (Hypothesis 1). First, we have regulated business interests that represent industries which are directly and profoundly targeted by agency regulations. This stakeholder type is our main reference category. We have three other types that are distinct from regulated businesses. To begin with, there are non‐regulated business interests, which concern industries that are not directly regulated and/or for which the regulated sector does not constitute their core economic activities, for instance, businesses depending on regulated industries. In order to simplify our work, we coded each stakeholder on the basis of the United Nations International Standard Industrial Classification of All Economic Activities (ISIC) assigning each business actor into an economic sector (United Nations, 2008; for a similar strategy see Young and Pagliari, 2017). First, for each agency, we checked all ISIC codes falling into the scope of the sector regulated by this agency. For the ECHA, for instance, these codes cover the manufacturing of chemicals and chemical products. Second, again for each agency, all business interests were coded as either a regulated sector (e.g. chemicals companies in the case of the ECHA) or a non‐regulated sector (e.g. car manufacturers in the case of the ECHA). Third, based on information provided on the organizational website, we cross‐checked the final coding for each stakeholder.

In addition to these business interests, we identified non‐business interests, which include organizations that do not represent companies but which do represent some general citizen interest. These cover consumer interests, specific causes such as human rights, animal rights, labour rights and environmental or health concerns, and other general causes. More specifically, within this category we find labour unions as well as a range of NGOs. For the purpose of this paper, modelling the involvement of non‐business interests is highly relevant because this actor type lacks resources and expertise, especially when compared to experts, other business interests, or public authorities. Finally, we identified a set of consultees that we label as ‘experts’, namely, professional associations, institutions such as hospitals or research institutes, and various national expert bodies.

## Results

**Figure 1:**
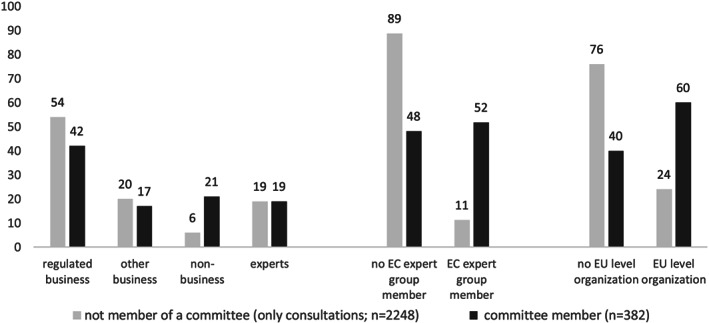
Closed consultation access compared across a) group type, b) EC expert group membership, and c) level of mobilization (percentages).

Before moving to the regression analysis, the distribution of the three main independent variables – group type, EC expert group membership, and level of mobilization – is contrasted with our dependent variable measuring whether a stakeholder was involved in an open consultation (0) or whether it also enjoyed access to closed consultations (1). The left part of Figure 1 shows that business interests, especially regulated industries, predominate both in public and closed consultations. However, non‐business interests are quite well represented in committees, in contrast to the limited participation in public consultations. Non‐business interests make up 21% of the committee members, but only 6% of the mobilized actors during public consultations. The right part of Figure 1 indicates that EC expert group members and EU‐level organizations are strongly represented in closed consultations, especially if we compare this with public consultations.

Table 1 confirms that open and closed consultations differ substantially. More precisely, non‐business interests and other business interests are generally more visible in committees, while compared to their participation in public consultations, regulated business interests are on average less prominent in the committees. The differences are less clear‐cut for experts. The number of experts that do not represent companies or business interests – for instance, in the case of the EFSA and EMA – may attenuate the prevalence of regulated businesses because the experts' professional activities could create conflicts of interest, for instance, because they depend on regulated companies for research grants (Corporate Europe Observatory, 2013). Despite this caveat, the descriptive analysis demonstrates that the distribution of stakeholders appearing in public consultations differs considerably from that of committee members.

**Table 1:** Share of non‐state stakeholder participation in open and closed consultations (percentages)

	open consultations					closed consultations					
	Regulated Business	Other Business	Non‐business	Experts	N	Regulated Business	Other Business	Non‐business	Experts	N
European Banking Authority (EBA)	77%	14%	2%	7%	208	37%	21%	23%	19%	43
European Securities and Markets Authority (ESMA)	60%	26%	3%	11%	466	33%	33%	14%	21%	58
European Insurance and Occupational Pensions Authority (EIOPA)	66%	21%	3%	11%	218	39%	12%	19%	30%	57
European Medicines Agency (EMA)	50%	10%	2%	38%	206	7%	5%	45%	43%	44
European Railway Agency (ERA)	43%	14%	43%	0%	7	75%	8%	17%	0%	12
European Food Safety Authority (EFSA)	24%	17%	16%	43%	519	49%	10%	25%	19%	51
European Aviation Safety Agency (EASA)	65%	8%	11%	19%	208	72%	0%	16%	12%	25
Agency for the cooperation of Energy regulators (ACER)	89%	9%	1%	1%	182	76%	18%	2%	4%	49
European Chemicals Agency (ECHA)	44%	51%	2%	3%	234	28%	33%	30%	9%	43
N	1209	459	143	437	2248	161	66	82	73	382

To test whether these bivariate results still hold when other independent variables are controlled for, we turn to the results of a multilevel logistic regression analysis. Since the units of analysis are clustered within nine EU agencies, it is likely that the assumptions of ordinary regression are violated. Stakeholders from the same agency can be expected to be more alike than what we would expect from a random sample, which may lead to biased standard errors and increases the risk of Type‐I errors, namely, finding a relationship where there is none. A multilevel analysis corrects this and models dependence and heterogeneity. To assess whether there are significant differences between agencies, a single‐level model without independent variables is compared to a model including only random intercepts for the agencies. A likelihood ratio test indicates a better fit for a multilevel modelling approach, taking into account variation between agencies (Δ*G*2 = −2*LL* without parameters−(−2*LL* random intercept model) = 32.96). As we have repeated observations for 264 organizations mobilizing towards two or more agencies, we report robust standard errors to account for dependence in the data.

We controlled for a number of other variables, namely organizational age and staff resources. Staff resources point at the capacity to acquire relevant expertise and the ability to monitor the policy process, affecting committee access in a positive way (Eising, 2007; Rasmussen and Gross, 2015). We measured staff resources by calculating z‐scores separately for individual firms, membership organizations, and institutions. The organizational age, measured as the natural logarithm of the number of years an organization has existed, can be seen as a proxy for reputation and is expected to affect access positively (Fraussen and Beyers, 2015; Furlong, 1997; Mahoney, 2004). The results reported in Table 2 do not include these control variables, since the likelihood ratio tests indicate that adding them does not substantially improve the model fit (Δ*G*2 = −2*LL* control variables –(−2*LL* model without controls) = 1.79; Table A2 Online Appendix). Neither organizational age nor staff significantly affect access to closed consultations. However, due to the lack of data on staff resources for many organizations, including these controls lead to a substantial reduction in the number of observations (*n* = 1825 versus *n* = 2630). Therefore, we should remain cautious when interpreting this result.

**Table 2:** Multilevel Logistic Regression Explaining Closed Consultation Access

	Model 1 all agencies	Model 2 agencies without legal provisions	Model 3 agencies with legal provisions	Model 4 all agencies, interaction group type*expert group
*Fixed Part*				
Intercept	−2.88 (0.32)***	−2.61 (0.50)*	−3.00 (0.41)***	−2.94 (0.37)***
Group type				
‐Regulated business (=ref.)	‐	‐	‐	‐
‐Other business	−0.07 (0.32)	−0.57 (0.60)	0.17 (0.33)	0.11 0.26)
‐Non‐business	0.60 (0.41)**	1.07 (0.90)	1.77 (0.61)**	1.61 0.48)***
‐Experts	−0.07 (0.32)	0.65 (0.60)	0.85 (0.47)	0.87 (0.40)*
EC Expert Group (ref. = No)	1.71 (0.28)***	2.22 (0.70)**	1.54 (0.29)***	1.87 (0.10)***
EU‐level organization (ref. = No)	1.11 (0.32)***	0.74 (0.32)*	1.27 (0.35)**	1.12 (0.32)***
Interaction: group type*expert group				
‐EC expert group/other business				−0.41 (0.21)
‐EC expert group/non‐business				−0.04 (0.42)
‐EC expert group/experts				−0.36 (0.42)
*Random Part*				
Agency‐level variance	0.30 (0.12)**	0.16 (0.07)*	0.37 (0.21)*	0.31 (0.12)**
*Model fit*				
Agencies	9	3	6	9
Actors	2630	739	1891	2630
AIC	1734.18	521.22	1216.49	1738.24
‐2LL	1720.18	507.22	1202.49	1718.24

Notes: coefficients are logits; robust standard errors in parentheses; * *p* < 0.05 ** *p* < 0.01 *** *p* < 0.001

Model 1 in Table 2 depicts the results for all nine agencies included and is the main focus of the discussion below. Models 2 and 3, respectively, present separate analyses for the six agencies with legal requirements (EMA, EBA, ESMA, EIOPA, EFSA, and ERA) and for the three agencies without legal requirements (ACER, EASA, and ECHA) to control for whether references to non‐business stakeholders in founding regulations matter.

**Figure 2:**
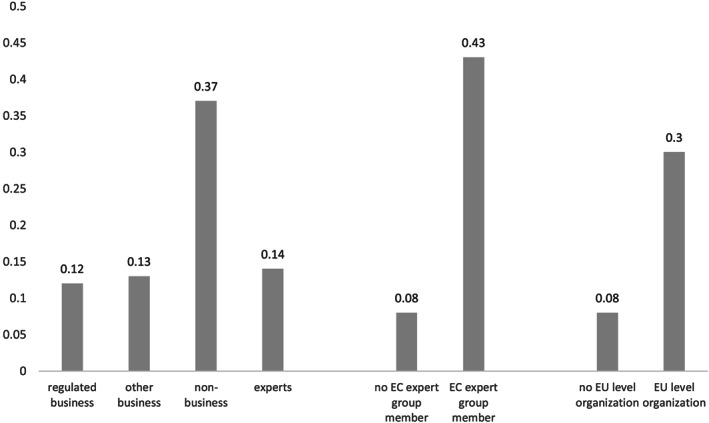
Predicted probability of closed consultation access by a) group type, b) EC expert group membership and c) level of mobilization (based on Model 1, N = 2630).

While Figure 1 shows that business interests – regulated and non‐regulated combined – dominate committees in absolute numbers, the regression results indicate that they do not necessarily gain more access compared to their share in the set of interests taking part in public consultations. There are no significant differences between groups representing the regulated industry and other business interests, but non‐business interests are quite well represented. Figure 2 shows the predicted probabilities of membership for the main organization types, keeping all other variables at their mean (based on Model 1). The probability of gaining access is 0.37 (S.E. = 0.27) for non‐business interests, compared with 0.12 (S.E. = 0.13) and 0.13 (S.E. = 0.14) respectively for regulated business and other business interests. Importantly, the probability that non‐business interests are selected for closed consultations should be understood in relation to their share in the public consultations. More precisely, out of the 225 identified non‐business interests, 36%, or 82 organizations, have a seat in a committee. There are twice as many regulated business committee representatives, namely 161, which is small (13%) compared to the entire set of mobilized regulated business interests in the public consultations (*n* = 1370).

A closer look at Model 2 and 3 is useful to assess the impact of explicit legal provisions on stakeholder involvement. Model 2 indicates that for agencies with such provisions there are no substantial differences for group type relative to the group type's share in the population of consultation participants. This is an interesting result as it implies that even in the absence of a legal provision with respect to the composition of committees, agency officials seek to ensure that non‐business groups gain access that is proportional to their mobilization in public consultations. Yet, we need to be careful with Model 2 as the evidence is limited to three agencies only. More interesting is Model 3 on six agencies with legal provisions. For these agencies, non‐business interests show a high likelihood of being selected, suggesting that the explicit provisions of stakeholder participation founding regulations may positively affect the selection for closed consultations.

Generally, these results contradict the common expectation that business interests have an almost natural advantage in gaining access to policymakers (Fraussen and Beyers, 2015; Moss and Carpenter, 2014; Rasmussen and Gross, 2015; Stigler, 1971). Rejecting the null‐hypothesis, however, demonstrates that consultation form matters and that representation in EU agency policymaking is also demand‐driven. Agency officials have some leeway in balancing interest representation and use their discretion to counterbalance the structural advantages of regulated business interests. Considerations other than the need for specialized information, largely supplied by regulated industries, play a role when agencies decide to whom they will grant access. This observation is in line with earlier findings that facilitating a balanced interest representation is an important motivation for establishing committees (Arras and Braun, 2018). As one agency official said during one of our interviews:

‘It is interesting that they offer more the other side of the coin, not just the industry side which we normally get through the normal stakeholders, but we get a consensus opinion on a topic.’ (EU agency official, 20/04/2015).

If we examine characteristics other than group type, EC expert group access is a strong predictor for closed consultation access. Figure 2 shows that the likelihood of access increases from 8% (S.E. = 0.07) to 43% (S.E. = 0.20) if a stakeholder belongs to an EC expert group. This result reflects earlier studies demonstrating the cumulative advantage of access (Binderkrantz et al., 2015). EU agencies, as relatively new institutions, not only provide additional access points to interested stakeholders but also reinforce the existing insider status some organized interests enjoy. One could imagine that some interest group types, for instance business interests, profit more from their insider position. Therefore, we controlled for the potential interaction between group type and EC expert group membership to see whether this cumulative mechanism works differently for some group types (Model 4). The non‐significant interaction, however, demonstrates that the cumulative mechanism of access does not disadvantage non‐business groups. Insiderness in one arena (for instance EC expert groups) thus leads to insiderness elsewhere (in this case, closed consultations organized by EU agencies), regardless of which interest is represented. Finally, EU‐level organizations also enjoy more access, indicating that representativeness is an important driver for closed consultations. Figure 2 shows that estimated probability of access rises from 0.08 (S.E. = 0.09) to 0.30 (S.E. = 0.21) for EU‐level organizations. This result is in line with the expectation that agency‐stakeholder interactions are used not only to acquire stakeholders' technical expertise but also to benefit from their organizational capacity and intermediary function (Arras and Braun, 2018; Braun, 2012/2013; Poppelaars, 2007). As one agency official said about their committee members:

‘We see them as very important and efficient multipliers. Because when you are located here in [agency location] and there are thousands and thousands of companies and stakeholders, speaking different languages and interested in different aspects, then there is no way ever that we would be able to reach out to all of them.’ (EU agency official, 23/03/2015).

## Conclusion

In this article, we compared closed consultations – who gains access to EU agency committees – with participation in public consultations. Theoretically, we started with the agency's motivations for interacting with stakeholders via committees. Expert information and organizational capacity were expected to increase access for groups who represent regulated industries, members of EC expert groups, and EU‐level associations. Alternatively, the agencies' need for reputation as an autonomous authority predicts the balancing of interest representation to ensure that interests other than the regulated industry obtain access. Hence, agencies use their discretion to diversify interest representation.

A first important finding is that while regulated business dominates closed consultations in absolute numbers, they have no higher chance of access than other interests in relative terms, namely, in comparison to their involvement in public consultations. Non‐business interests, such as NGOs and trade unions, have a considerable chance to obtain access. Compared with their relative share in the open consultations, these non‐business interests, which are expected to face more difficulties in mobilizing and gaining access to policymakers, are surprisingly well represented (Dür and Matteo, 2016; Olson, 1965; Rasmussen and Gross, 2015). This finding indicates that committees not only serve a need for expertise supplied by regulated industries but are also used to signal an attempt to balance interest representation, diversify the supply of information, and mitigate allegations of sole dependence on regulated business interests (Carpenter and Moss, 2014; Gornitzka and Sverdrup, 2015). This fits within a reputational view of agency autonomy, where interactions with a diverse group of organized interests can be strategically used to prevent dependence on a single subset of societal interests and acquire more legitimacy (Carpenter, 2001/2010; Moffitt, 2010).

This result also illustrates the power of institutions in steering interest representation (Mahoney, 2004). The three financial regulatory agencies (EBA, ESMA, and EIOPA) are an interesting example of how biased interest representation can be compensated for and how some diversity is achieved by combining open and closed consultation forms. While more than 80% of the mobilized stakeholders on financial regulation represent business interests, and public interest groups such as consumer organizations hardly ever participate in these consultations, the closed consultations of these three agencies are the most diverse of all, with about half of the seats reserved for experts and non‐business interests (Pagliari and Young, 2016). When establishing these agencies, the legislator – in this case the European Parliament and the Council – aimed to institutionalize some balanced interest representation as their legal statutes include explicit provisions regarding the representation of the financial industry, consumers, trade unions, and academics (see Table A1).

A second important result is that groups that already enjoy an insider status as members of EC expert groups are more likely to obtain access to EU agencies. In fact, about half of the committee members also hold a seat in an EC expert group. Hence, there exists a group of organized interests with a core insider status that have access at both the early legislative stage of the policymaking process and during the implementation stage. Therefore, on the one hand, EU agencies can foster diversity in their stakeholder community by steering who gains access to committees. On the other hand, most committee seats are occupied by actors that have already obtained access to the earlier stages of the policy process (Binderkrantz et al., 2015). There could be an omitted variable explaining access to both EC expert groups and EU agency committees, namely, the ability to provide expertise. Nonetheless, group resources do not improve access significantly.

In short, while business interests clearly dominate in absolute numbers, institutionalized interest representation in EU regulatory agencies is not as biased towards regulated business as capture theory would expect, and agencies clearly try to avoid such bias. Yet, follow‐up research needs to address some important remaining questions.

First, committees ensure the representation of alternative voices in terms of other business as well as non‐business interests and such a more balanced representation may foster the input of various policy ideas. Yet, this in itself does not guarantee that different viewpoints are effectively heard. Research on interest group mobilization in the financial sector, for instance, shows that mobilized groups from outside the financial sector often express similar preferences to those of the financial sector, meaning that in this case, other business interests do not necessarily constitute a counter voice to the regulated industry (Pagliari and Young, 2016; Young and Pagliari, 2017; see also Chalmers, 2015). Hence, the question remains to what extent variation in group type reflects different policy preferences with respect to concrete regulatory policies. Finally, the use of various consultation devices – such as public consultations and committees – is based on the presumption that engaging stakeholders will increase the legitimacy of policymaking procedures and lead to higher quality policy outputs (Agné et al., 2015; Bernauer and Gampfer, 2013; Kohler‐Koch and Quittkat, 2013; Tyler, 2006). However, the question remains whether the modest efforts to balance and diversify interest representation effectively contribute to the agencies' reputational autonomy and legitimacy. Therefore, future research should examine to what extent the presence of non‐regulated interests is just a signal of diversity and autonomy, or whether this actually leads to policies that do not systematically favour regulated industries.

## Supporting information


**Table S1.** Stakeholder Participation Mentioned in Founding Regulations of EU Agencies
**Table S2.** Multilevel Logistic Regression Explaining Advisory Committee Membership, control variables staff resources and organizational age

## References

[jcms12991-bib-0001] Agné, H. , Dellmuth, L.M. and Tallberg, J. (2015) ‘Does stakeholder involvement foster democratic legitimacy in international organizations? An empirical assessment of a normative theory’. The Review of International Organizations, Vol. 10, No. 1, pp. 465–488.

[jcms12991-bib-0002] Arras, S. and Braun, C. (2018) ‘Stakeholders wanted! Why and how European Union agencies involve non‐state stakeholders’. Journal of European Public Policy, Vol. 25, No. 9, pp. 1257–1275.

[jcms12991-bib-0003] Barkow, R.E. (2010) ‘Insulating Agencies: Avoiding Capture Through Institutional Design’, *New York University Public Law and Legal Theory Working Papers* (Paper 240).

[jcms12991-bib-0004] Bernauer, T. and Gampfer, R. (2013) ‘Effects of civil society involvement on popular legitimacy of global environmental governance’. Global Environmental Change, Vol. 23, No. 2, pp. 439–449.

[jcms12991-bib-0005] Beyers, J. and Arras, S. (2019) ‘Who feeds information to regulators? Diversity in stakeholder participation in European Union regulatory agency consultations’. Journal of Public Policy. 10.1017/S0143814X19000126.

[jcms12991-bib-0006] Binderkrantz, A.S. , Christiansen, P.M. and Pedersen, H.H. (2015) ‘Interest group access to the bureaucracy, parliament, and the media’. Governance, Vol. 28, No. 1, pp. 95–112.

[jcms12991-bib-0007] Binderkrantz, A.S. , Pedersen, H.H. and Beyers, J. (2016) ‘What is access? A discussion of the definition and measurement of interest group access’. European Political Science, Vol. 16, No. 3, pp. 306–321.

[jcms12991-bib-0008] Borrás, S. , Koutalakis, C. and Wendler, F. (2007) ‘European Agencies and Input Legitimacy: EFSA, EMeA and EPO in the Post‐Delegation Phase’. Journal of European Integration, Vol. 29, No. 5, pp. 583–600.

[jcms12991-bib-0009] Bouwen, P. (2002) ‘Corporate lobbying in the European Union: the logic of access’. Journal of European Public Policy, Vol. 9, No. 3, pp. 365–390.

[jcms12991-bib-0010] Braun, C. (2012) ‘The Captive or the Broker? Explaining Public Agency‐Interest Group Interactions’. Governance, Vol. 25, No. 2, pp. 291–314.

[jcms12991-bib-0011] Braun, C. (2013) ‘The Driving Forces of Stability Exploring the Nature of Long‐Term Bureaucracy–Interest Group Interactions’. Administration and Society, Vol. 45, No. 7, pp. 809–836.

[jcms12991-bib-0012] Bunea, A. and Thomson, R. (2015) ‘Consultations with Interest Groups and the Empowerment of Executives: Evidence from the European Union’. Governance, Vol. 28, No. 4, pp. 517–531.

[jcms12991-bib-0013] Busuioc, E.M. and Lodge, M. (2016) ‘The Reputational Basis of Public Accountability’. Governance, Vol. 29, No. 2, pp. 247–263.

[jcms12991-bib-0014] Carpenter, D.P. (2001) The Forging of Bureaucratic Autonomy: Reputations, Networks, and Policy Innovation in Executive Agencies, 1862–1928 (Princeton: Princeton University Press).

[jcms12991-bib-0015] Carpenter, D.P. (2010) Reputation and Power: Organizational Image and Pharmaceutical Regulation at the FDA (Princeton: Princeton University Press).

[jcms12991-bib-0016] Carpenter, D.P. and Moss, D.A. (2014) Preventing Regulatory Capture: Special Interest Influence and How to Limit it (Cambridge: Cambridge University Press).

[jcms12991-bib-0017] Chalmers, A.W. (2015) ‘Financial industry mobilisation and securities markets regulation in Europe’. European Journal of Political Research, Vol. 54, No. 3, pp. 482–501.

[jcms12991-bib-0018] Coglianese, C. , Zeckhauser, R. and Parson, E.A. (2004) ‘Seeking Truth for Power: Informational Strategy and Regulatory Policy Making’. Minnesota Law Review, Vol. 89, No. 2, pp. 277–341.

[jcms12991-bib-0019] Corporate Europe Observatory (2013) *Unhappy meal. The European Food Safety Authority's independence problem*. goo.gl/CZT5Zw (accessed on 8 February 2019).

[jcms12991-bib-0020] De Bruycker, I. (2016) ‘Pressure and Expertise: Explaining the Information Supply of Interest Groups in EU Legislative Lobbying’. JCMS, Vol. 54, No. 3, pp. 599–616.

[jcms12991-bib-0021] Dür, A. and Matteo, G. (2016) Insiders versus outsiders: Interest group politics in multilevel Europe (Oxford: Oxford University Press).

[jcms12991-bib-0022] EFSA (2015) ‘Conclusion on the peer review of the pesticide risk assessment of the active substance glyphosate’. EFSA Journal, Vol. 13, No. 11, p. 4302. 10.2903/j.efsa.2015.4302 PMC13189512 42169844

[jcms12991-bib-0023] Egeberg, M. and Trondal, J. (2017) ‘Researching European Union Agencies: What Have We Learnt (and Where Do We Go from Here)?’ JCMS, Vol. 55, No. 4, pp. 675–690.

[jcms12991-bib-0024] Eising, R. (2007) ‘Institutional Context, Organizational Resources and Strategic Choices: Explaining Interest Group Access in the European Union’. European Union Politics, Vol. 8, No. 3, pp. 329–362.

[jcms12991-bib-0025] Fraussen, B. and Beyers, J. (2015) ‘Who's in and who's out?: Explaining access to policymakers in Belgium’. Acta Politica, Vol. 51, No. 1, pp. 214–236.

[jcms12991-bib-0026] Fraussen, B. , Beyers, J. and Donas, T. (2014) ‘The Expanding Core and Varying Degrees of Insiderness: Institutionalised Interest Group Access to Advisory Councils’. Political Studies, Vol. 63, No. 3, pp. 569–588.

[jcms12991-bib-0027] Furlong, S.R. (1997) ‘Interest Group Influence on Rule Making’. Administration and Society, Vol. 29, No. 3, pp. 325–347.

[jcms12991-bib-0028] Furlong, S.R. and Kerwin, C.M. (2005) ‘Interest Group Participation in Rule Making: A Decade of Change’. Journal of Public Administration Research and Theory, Vol. 15, No. 3, pp. 353–370.

[jcms12991-bib-0029] Gornitzka, Å. and Sverdrup, U. (2011) ‘Access of Experts: Information and EU Decision‐making’. West European Politics, Vol. 34, No. 1, pp. 48–70.

[jcms12991-bib-0030] Gornitzka, Å. and Sverdrup, U. (2015) ‘Societal Inclusion in Expert Venues: Participation of Interest Groups and Business in the European Commission Expert Groups’. Politics & Governance, Vol. 3, No. 1, pp. 151–165.

[jcms12991-bib-0031] Grossmann, M. (2012) The Not‐So‐Special Interests: Interest groups, public representation and American governance (Stanford: Stanford University Press).

[jcms12991-bib-0032] Halpin, D. (2011) ‘Explaining Policy Bandwagons: Organized Interest Mobilization and Cascades of Attention’. Governance, Vol. 24, No. 2, pp. 205–230.

[jcms12991-bib-0033] Hannan, M.T. and Freeman, J. (1989) Organizational Ecology (Cambridge: Harvard University Press).

[jcms12991-bib-0034] Kelemen, D.R. (2002) ‘The Politics of “Eurocratic” Structure and the New European Agencies’. West European Politics, Vol. 25, No. 4, pp. 93–118.

[jcms12991-bib-0035] Kohler‐Koch, B. and Quittkat, C. (2013) De‐Mystification of Participatory Democracy: EU‐Governance and Civil Society (Oxford: Oxford University Press).

[jcms12991-bib-0036] Koop, C. (2014) ‘Theorizing and Explaining Voluntary Accountability’. Public Administration, Vol. 92, No. 3, pp. 565–581.

[jcms12991-bib-0037] Kwak, J. (2014) ‘Cultural Capture and the Financial Crisis’. In Carpenter D.P. and Moss D.A. (eds) Preventing Regulatory Capture: Special Interest Influence and How to Limit it (New York: Cambridge University Press), pp. 71–98.

[jcms12991-bib-0038] Mahoney, C. (2004) ‘The Power of Institutions: State and Interest Group Activity in the European Union’. European Union Politics, Vol. 5, No. 4, pp. 441–466.

[jcms12991-bib-0039] Majone, G. (1999) ‘The regulatory state and its legitimacy problems’. West European Politics, Vol. 22, No. 1, pp. 1–24.

[jcms12991-bib-0040] March, J.G. and Olsen, J.P. (1989) Rediscovering Institutions. The Organizational Basis of Politics (New York: The Free Press).

[jcms12991-bib-0041] Martinez, M.G. , Verbruggen, P. and Fearne, A. (2013) ‘Risk‐based approaches to food safety regulation: what role for co‐regulation?’ Journal of Risk Research, Vol. 16, No. 9, pp. 1101–1121.

[jcms12991-bib-0042] McCubbins, M.D. and Schwartz, T. (1984) ‘Congressional Oversight Overlooked: Police Patrols versus Fire Alarms*’. American Journal of Political Science, Vol. 28, No. 1, pp. 165–179.

[jcms12991-bib-0043] Merton, T.K. (1968) ‘The Matthew Effect in Science’. Science, Vol. 159, No. 3810, pp. 56–63. 5634379

[jcms12991-bib-0044] Moffitt, S.F. (2010) ‘Promoting Agency Reputation through Public Advice: Advisory Committee Use in the FDA’. Journal of Politics, Vol. 72, No. 3, pp. 880–893.

[jcms12991-bib-0045] Molina, O. and Rhodes, M. (2002) ‘Corporatism: The Past, Present, and Future of a Concept’. Annual Review of Political Science, Vol. 5, No. 1, pp. 305–331.

[jcms12991-bib-0046] Moss, D.A. and Carpenter, D.P. (2014) ‘Conclusion’. In Carpenter D.P. and Moss D.A. (eds) Preventing Regulatory Capture: Special Interest Influence and How to Limit it (New York: Cambridge University Press), pp. 451–465.

[jcms12991-bib-0047] Olson, M. (1965) The Logic of Collective Action: Public Goods and the Theory of Groups (Cambridge: Harvard University Press).

[jcms12991-bib-0048] Ottow, A.T. (2015) Market and Competition Authorities. Good Agency Principles (Oxford: Oxford University Press).

[jcms12991-bib-0049] Pagliari, S. and Young, K.L. (2014) ‘Leveraged interests: Financial industry power and the role of private sector coalitions’. Review of International Political Economy, Vol. 21, No. 3, pp. 575–610.

[jcms12991-bib-0050] Pagliari, S. and Young, K.L. (2016) ‘The interest ecology of financial regulation: Interest group plurality in the design of financial regulatory policies’. Socio‐Economic Review, Vol. 14, No. 2, pp. 309–337.

[jcms12991-bib-0051] Pedersen, H.H. , Halpin, D.R. and Rasmussen, A. (2015) ‘Who Gives Evidence to Parliamentary Committees ? A Comparative Investigation of Parliamentary Committees and their Constituencies’. Journal of Legislative Studies, Vol. 21, No. 3, pp. 408–427.

[jcms12991-bib-0052] Pérez Durán, I. (2018) ‘Interest group representation in the formal design of European Union agencies’. Regulation & Governance, Vol. 12, No. 2, pp. 238–262.

[jcms12991-bib-0053] Pérez Durán, I. (2019) ‘Political and stakeholder's ties in European Union agencies’. Journal of European Public Policy, Vol. 26, No. 1, pp. 1–22.

[jcms12991-bib-0054] Poppelaars, C. (2007) ‘Resource Exchange in Urban Governance: On the Means that Matter’. Urban Affairs Review, Vol. 43, No. 1, pp. 3–27.

[jcms12991-bib-0055] Quittkat, C. (2011) ‘The European Commission's Online Consultations: A Success Story?’ JCMS, Vol. 49, No. 3, pp. 653–674.

[jcms12991-bib-0056] Quittkat, C. and Kotzian, P. (2011) ‘Lobbying via Consultation ‐ Territorial and Functional Interests in the Commission's Consultation Regime’. Journal of European Integration, Vol. 33, No. 4, pp. 401–418.

[jcms12991-bib-0057] Rasmussen, A. (2015) ‘Participation in Written Government Consultations in Denmark and the UK: System and Actor‐level Effects’. Government and Opposition, Vol. 50, No. 2, pp. 271–299.

[jcms12991-bib-0058] Rasmussen, A. and Gross, V. (2015) ‘Biased access? Exploring selection to advisory committees’. European Political Science Review, Vol. 7, No. 3, pp. 343–372.

[jcms12991-bib-0059] Selznick, P. (1948) ‘Foundations of the Theory of Organization’. American Sociological Review, Vol. 13, No. 1, pp. 25–35.

[jcms12991-bib-0060] Stigler, G. (1971) ‘The Theory of Economic Regulation’. Bell Journal of Economics and Management Science, Vol. 2, No. 1, pp. 3–21.

[jcms12991-bib-0061] Thiel, M. (2014) ‘European Civil Society and the EU Fundamental Rights Agency: Creating Legitimacy through Civil Society Inclusion?’ Journal of European Integration, Vol. 36, No. 5, pp. 435–451.

[jcms12991-bib-0062] Tyler, T.R. (2006) ‘Psychological Perspectives on Legitimacy and Legitimation’. Annual Review of Psychology, Vol. 57, pp. 375–400. 10.1146/annurev.psych.57.102904.190038 16318600

[jcms12991-bib-0063] United Nations (2008) *International Standard Industrial Classification of All Economic Activities* (Series M No. 4/Rev.4). Department of Economic and Social Affairs, Statistics Department, goo.gl/8dw5k3 (accessed 8 February 2019).

[jcms12991-bib-0064] Van Ballaert, B. (2017) ‘The European Commission's use of consultation during policy formulation: The effects of policy characteristics’. European Union Politics, Vol. 18, No. 3, pp. 406–423.

[jcms12991-bib-0065] Verbruggen, P. (2013) ‘Gorillas in the closet? Public and private actors in the enforcement of transnational private regulation’. Regulation & Governance, Vol. 7, No. 4, pp. 512–532.

[jcms12991-bib-0066] Wonka, A. and Rittberger, B. (2010) ‘Credibility, complexity and uncertainty: explaining the institutional independence of 29 EU agencies’. West European Politics, Vol. 33, No. 4, pp. 730–752.

[jcms12991-bib-0067] Yackee, J.W. and Yackee, S.W. (2006) ‘A Bias Towards Business? Assessing Interest Group Influence on the U.S. Bureaucracy’. The Journal of Politics, Vol. 68, No. 1, pp. 128–139.

[jcms12991-bib-0068] Yackee, S.W. (2014) ‘Participant Voice in the Bureaucratic Policymaking Process’. Journal of Public Administration Research and Theory, Vol. 25, No. 2, pp. 427–449.

[jcms12991-bib-0069] Young, K.L. and Pagliari, S. (2017) ‘Capital united? Business unity in regulatory politics and the special place of finance’. Regulation & Governance, Vol. 11, No. 1, pp. 2–23.

