# Supplementary material for: Access to European Union Agencies: Usual Suspects or Balanced Interest Representation in Open and Closed Consultations?
Source: J Common Mark Stud. 2019 Dec 29;58(4):836–55. doi: 10.1111/jcms.12991 (PMC7386941; doi:10.1111/jcms.12991)
Supplement: Supplementary file 1 — Table S1. Stakeholder Participation Mentioned in Founding Regulations of EU Agencies Table S2. Multilevel Logistic Regression Explaining Advisory Committee Membership, control variables staff resources and organizational age [file JCMS-58-836-s001.docx]

**Table A1. Stakeholder Participation Mentioned in Founding Regulations of EU Agencies**

| Agency |  | Stakeholder types mentioned in the founding regulation |
| --- | --- | --- |
| European Medicines Agency (EMA) |  | - Patient organizations - Healthcare professionals |
| European Banking Authority (EBA) |  | - Credit and investment institutions, representing the diverse models and sizes of financial institutions and businesses, including, as appropriate, institutional investors and other financial institutions that themselves use financial services - Small and medium-sized enterprises (SMEs) - Trade unions - Academics - Consumers and other retail users of banking services |
| European Securities and Markets Authority (ESMA) |  | - Financial market participants - SMEs - Trade unions - Academics - Consumers and other retail users of financial services - Professional associations |
| European Insurance and Occupational Pensions Authority (EIOPA) |  | - Insurance and reinsurance undertakings and insurance intermediaries - SMEs - Trade unions - Academics - Consumers and other retail users of insurance and reinsurance services - Professional associations |
| European Food Safety Authority (EFSA) |  | - Consumer representatives - Producer representatives - Processors - Any other interested parties |
| European Railway Agency (ERA) |  | - The industry - Workers’ organizations |
| European Aviation Safety Agency (EASA) |  | / |
| European Chemicals Agency (ECHA) |  | / |
| Agency for the cooperation of Energy regulators (ACER) |  | / |

**Table A2. Multilevel Logistic Regression Explaining Advisory Committee Membership, control variables staff resources and organizational age**

|  | with control variables | without control variables |
| --- | --- | --- |
| *Fixed Part* |  |  |
| Intercept | -3.11 (0.42)*** | -2.83 (0.37)*** |
| Group type |  |  |
| Regulated business (=ref.) | - | - |
| Other business | -0.14 (0.27) | 0.12 (0.28) |
| Non-business | 1.96 (0.49)*** | 1.94 (0.49)*** |
| Experts | 0.97 (0.41)* | 0.99 (0.40)* |
| EC Expert Group (ref.=No) | 1.52 (0.31)*** | 1.56 (0.29)*** |
| EU-level organization (ref.=No) | 1.23 (0.28)*** | 1.16 (0.31)*** |
| Staff resources | 0.05 (0.09) |  |
| Organizational age | 0.08 (0.05) |  |
| *Random Part* |  |  |
| Agency-level variance | 0.40 (0.24)* | 0.38 (0.21)* |
| *Model fit* |  |  |
| Agencies | 9 | 9 |
| Actors | 1841 | 1841 |
| AIC | 1274.47 | 1272.26 |
| -2LL | 1256.47 | 1258.26 |

Note: coefficients are logits; robust standard errors in parentheses; * p< 0.05 ** p< 0.01 *** p< 0.001.
